# Effect of different milling methods on physicochemical and functional properties of mung bean flour

**DOI:** 10.3389/fnut.2023.1117385

**Published:** 2023-02-24

**Authors:** Shibo Yu, Yanchun Wu, Zhenjiang Li, Changyuan Wang, Dongjie Zhang, Lidong Wang

**Affiliations:** ^1^College of Food Science, Heilongjiang Bayi Agricultural University, Daqing, China; ^2^Department of National Coarse Cereals Engineering Research Center, Heilongjiang Bayi Agricultural University, Daqing, China; ^3^Quality Supervision, Inspection and Testing Center of Agricultural Processed Products Ministry of Agriculture, Heilongjiang Bayi Agricultural University, Daqing, China

**Keywords:** mung bean, ball mill, vibratory mill, physicochemical properties, functional properties

## Abstract

There needs to be more information concerning the effect of different milling methods on the physicochemical properties of whole-grain mung bean flour. Therefore, the physicochemical properties of whole grain mung bean flour were analyzed using universal grinders (UGMB), ball mills (BMMB), and vibration mills (VMMB). The results showed that the particle size of the sample after ultrafine grinding treatment was significantly reduced to 21.34 μm (BMMB) and 26.55 μm (VMMB), and the specific surface area was increased. The particle distribution was uniform to a greater extent, and the color was white after treatment. Moreover, the water holding capacity (WHC), oil holding capacity (OHC), and swelling power (SP) increased, and the bulk density and solubility (S) decreased. The Rapid Viscosity Analyzer (RVA) indicated that the final viscosity of the sample after ultrafine grinding was high. Furthermore, rheological tests demonstrated that the consistency coefficient K, shear resistance, and viscosity were decreased. The results of functional experiments showed that the treated samples (BMMB and VMMB) increased their capacity for cation exchange by 0.59 and 8.28%, respectively, bile acid salt adsorption capacity increased from 25.56 to 27.27 mg/g and 26.38 mg/g, and nitrite adsorption capacity increased from 0.58 to 1.17 mg/g and 1.12 mg/g.

## 1. Introduction

Evidence from current scientific dietary interventions and metabolic studies supports the concept that whole grain foods and grain legumes have functional properties that protect against metabolic disorders and reduce the risk of chronic diseases, such as Type II diabetes, cardiovascular disease, and body weight management (1–3). Grain legumes, such as chickpeas, lentils, peas, black beans, navy beans, mung bean and cowpea beans, are rich sources of proteins, complex carbohydrates, dietary fibers, vitamins, minerals, and other bioactive components such as polyphenols and flavonoids. These nutrient components could contribute to legumes’ health benefits and prevent diseases (4, 5). Therefore, legumes have recently gained more attention as ingredients (product formulators and consumers seek) in daily diets and the food industry to improve nutritional quality. However, traditional foods such as noodles, steamed bread, pasta, baked goods, and snacks, which are predominantly made from cereal flours such as wheat, maize, or rice flour, are starch-rich and lack essential nutrients such as dietary fiber, vitamins, a balanced amino acid diet, and bioactive substance, and these substances are mainly attributed to the refinement of production and processing (6, 7).

Mung bean (*Vigna radiate* L.), known as green gram, is an important edible traditional grain legume consumed by most Asian countries (8). Mung bean has proven to be a good source of protein (rich in lysine, leucine, and threonine), dietary fiber, resistant starch, minerals, and in combination with other beneficial health components, such as phenolic compounds and polysaccharides (9). As a nutritional and healthy ingredient, mung bean flour is commonly prepared into various products such as noodles, bread, snacks, and vermicelli (2). In order to get better qualities and blend well of the flours, some processing techniques such as germination (10), extrusion (7), and common grinding (11) have been used.

Displacing all or part of the cereals from these products to imbue them with the healthful attributes of legumes requires the excellent miscibility of legume ingredients. Milling is a primary and commonly used method that can reduce the size of legumes to a greater extent and allows legume ingredients to be blended well (7). Size reduction is a major unit operation in various industries and significantly affects the chemical composition (ash, protein, and starch content) and nutritional, functional, and technological properties (12, 13, 14). There are three main milling methods: wet, semi-dry, and dry milling. Due to high costs and environmental concerns, the dry milling method is frequently used, and the usual machinery for milling includes the ball, hammer, pin, blade, and jet mills (15). The different types of mills and grinding methods significantly affect the physicochemical and functional properties of flours, such as damaged starch, polyphenol content, swelling power, and pasting properties (16–19).

As innovative technologies, vibration milling and planetary ball milling are considered the most effective methods to prepare ultrafine powder and have been widely used in the pharmaceutical and material industries (16, 20). However, only a few advances were made in food development. Vibratory mill grinding is a high-speed grinding technology that processes powder into microparticles. Grinding is done in the crushing chamber by cutting, grinding, and friction between high-strength grinding rods. In addition, it also has the advantages of a large feeding capacity and continuous production. Recent studies in the food system have focused on the milling of cereal and legume flours using a vibratory mill and its effect on the properties of micronized flours from germinated brown rice (21), carob (22), barley and rye (23), wheat (16, 19, 24). However, planetary ball milling is used to grind and mix samples at high speed and make the planetary motion, which refers to friction, collision, impingement, shear, or other mechanical actions to modify the properties of the samples (24). This technology can improve material characteristics without producing hazardous materials. Ball milling treatment was seldom used in food processing, especially the grinding of cereal and legume flours. Only a few reports have shown that ball milling was applied to improve the physicochemical and morphological properties of sorghum flour (20), thermal and rheological properties of oat bran protein flour (25), functional properties of extruded chickpea powder (18) and the thermomechanical properties of rice flour (14).

However, limited studies have been published on the effect of different milling methods on mung bean flour’s physiochemical and functional properties. The objective of this study was to apply different milling methods, including vibration milling, ball milling, and common grinding, to prepare mung bean flour and observe their influences on the properties of flour.

## 2. Materials and methods

### 2.1. Materials

Coarse mung bean powder (UGMB) was prepared from whole seed mung beans with a moisture content of 7% by crushing machine. The mung bean variety, Bahaxibo, was purchased from Daqing City, Heilongjiang Province, China. Mung beans were cleaned prior to coarse crushing (free from possible dust, grains, stones, insects etc.), All chemical reagents used in this study were of analytical grade.

### 2.2. Raw material treatment

#### 2.2.1. Preparation of mung bean flour by ball milling

Coarsely crushed mung bean was ground using ball mill (QM-ISP2, Nanjing University Instrument Factory, Nanjing City, Jiangsu Province, China.) for 35 h to get mung bean superfine powder (BMMB). During the milling process, the speed of 455 rpm. The ratio of spheres to material was 3:1, and the ratio of large to small spheres in agate balls was 1:4.

#### 2.2.2. Preparation of mung bean powder by vibrating mill

Coarsely crushed mung bean was ground using vibratory mill (JFM-50, Jinan Jianchen Machinery Co, Jinan City, Shandong Province, China.) for 25 min to obtain mung bean superfine powder (VMMB).

### 2.3. Powder properties

#### 2.3.1. Particle size and distribution

The particle-size distribution of mung bean flour was determined with a laser particle size analyzer (Bettersize2000, Dandong Baite Instrument Co, Dandong City, Liaoning Province, China.), where distilled water was used as a dispersant.

#### 2.3.2. Scanning electron microscopy

The mung bean samples were fixed to SEM stubs with double adhesive tape and coated with gold. The micrographs of mung bean samples were obtained using a scanning electron microscopy (SU8020, Hitachi Japan, Tokyo, Japan.). Set the SEM parameters: magnification 1,000x, voltage 3.0 kV, vacuum 6E.0 Pa. SEM morphological images were taken under this condition.

#### 2.3.3. Particle density

Taked 15 g(m) of mung bean powder into a measuring cylinder, and the measuring cylinder was fixed on the vibration density meter (MZ-3003, Seconds Technology (Shenzhen) Co, Shenzhen City, Guangdong Province, China.), where the stroke was 3 mm and number 1000 was selected until the scale in the measuring cylinder did not drop. The volume (*V*) of the sample were recorded and the vibrational density was expressed as *m*/*V* (g/mL). A certain amount mung bean was put into a natural bulk density meter [MZ-103, Seconds Technology (Shenzhen) Co, Shenzhen City, Guangdong Province, China], allowing the sample to fall naturally until the container below the instrument was filled. The volume (*V*) and weight (*m*) of the sample were recorded and the stacking density was expressed as *m*/*V* (g/mL) (26).

#### 2.3.4. Measurement of color

Place the colorimeter (CR-410, KONICA MINOLTA, Tokyo, Japan.) on the zeroing white board for zeroing according to the instructions for use. Place the sample under a white background plate, flatten and compact it, place the colorimeter on the sample, and measure the color of the sample. Each sample was measured three times. Color was determined using Cao et al. (27).

### 2.4. Analysis of physicochemical and functional properties

#### 2.4.1. Water and oil absorption capacity

Water absorption capacity (WAC) and oil absorption capacity (OAC) of mung beans were analyzed as described by Chanvorleak et al. (28) and Li et al. (29) with slight modifications.

In brief, 1 g of sample (m) was dispersed in 20 ml of water in a pre-weighed centrifuge tube (m_1_) using a vortex shaker and cooked at 60°C in a water bath for 30 min. The heated paste was cooled in an ice-water bath for another 30 min and then centrifuged at 4000 rpm for 20 min. The resulting supernatant was removed and the centrifuge tube (m_2_) with sediment was weighed again. The WAC was calculated using the following equation:


(1) $$\mathrm{WAC}\;\left(g/g\right)=\frac{m_{2}-m_{1}-m}{m}$$

Oil absorption capacity was determined by the same method as WAC. Two grams of sample (m) and 30 ml of oil were mixed for 5 min (for each 30s) in a pre-weighed centrifuge tube (m_1_) using a vortex mixer and centrifuged at 4,000 rpm for 20 min. The resulting supernatant was removed and the centrifuge tube (m_2_) with sediment was weighed again. The OAC was calculated using the following equation:


(2) $$\mathrm{OAC}\;\left(g/g\right)=\frac{m_{2}-m_{1}-m}{m}$$

#### 2.4.2. Solubility and swelling power (S and SP)

Solubility (S) and swelling power (SP) mung bean were measured following the method of Zhang et al. (30). With slight modifications. 1 g sample (m_1_) was made into starch emulsion (2%) and stirred in a water bath at 85°C for 30 min. The mixture was centrifuged at 4,000 rpm for 20 min. The supernatant was poured into an aluminum box (m_2_) with constant weight and dried to a constant weight (m_3_) at 105°C. Additionally, the precipitate was dried to a constant weight (m_4_). Each measurements were performed in duplicate. The S (%) and SP (g/g) were computed by the following formulas:


(3) $$S\;\left(\%\right)=\frac{m_{3}-m_{2}}{m_{1}}$$

(4) $$\mathrm{SP}\;\left(g/g\right)=\frac{m_{4}}{1-S}$$

### 2.5. Pasting properties

Pasting properties were evaluated using a rapid viscosity analyzer (RVA-20, Nantong Jinyu Bosin Only Technology Co, Nantong City, Jiangsu Province, China.), according to Yang et al. (31). The process was carried out using a 10% mung bean powder solution (distilled water as dispersant), the measurement method was performed using program 1: the initial stirring paddle was run at 960 r/min for 8 s, and then 160 r/min was kept stable. The temperature was ramped up from 48 to 95°C at 8.3°C/min and stabilized for 2.5 min. Then it was decreased from 95 to 49°C at 10.3°C/min and stabilized for 3.3 min. The pasting characteristics (peak viscosity, valley viscosity, final viscosity and pasting temperature) of the samples were measured under this procedure and repeated three times for each sample.

### 2.6. Thermal properties

The thermal properties of mung bean flour were analyzed using a synchronous thermal analyzer (TA Q200, TA United States, Newcastle City, Delaware, United States) as described by de Barros Mesquita et al. (32) with slight modifications.2 mg sample and 6 μl water were added to the aluminum box. Then the sample was sealed, kept at room temperature for 2 h and allowed to mix well. The sample tray was placed into the differential scanning calorimeter with a temperature and heating rate of room temperature to 110°C at 10°C/min, respectively.

### 2.7. Rheological properties

The rheological properties of mung bean flour were measured using rheometer [MCR92, Anton Paar (Shanghai) Trading Co, Shanghai City, China], referring to the method of Jiang et al. (33) with minor modifications. Mung bean powder solution with a concentration of 5% was prepared and pasted in a water bath at 90°C for 30 min. The treated samples were placed in a rheometer and the gap between the parallel plate and Peltier plate was adjusted to 1,000 μm, and the shear rate range was set to 0.01–100 1/s, the shear stress range to 1–200 Pa, the angular frequency to 0.1–100 rad/s, and the shear strain range to 0.001–10%. The samples were tested for viscosity, amplitude scan and frequency scan.

### 2.8. Functional characteristics

#### 2.8.1. Cation exchange capacity

Cation exchange capacity was evaluated by slightly modifying the method described by Hongcheng (34). Mung bean powder (0.5 g) and 100 ml 5% NaCl (*w*/*v*) were mixed in a beaker. The pH of the solution was calculated after stirring for 5 min (PHS-25, Shanghai Oshitol Industrial Co, Shanghai, China.). Afterward, 0.1 ml of 0.01 mol/l NaOH was added, and the pH was measured every 5 min until the total amount of NaOH was added to 1 ml.

#### 2.8.2. Cholate adsorption capacity

The cholate adsorption capacity of mung bean flour was determined according to the method of Xia et al. (35) with some modification. 30 ml of 1 mg/ml sodium cholate solution (pH = 7) with 1 g of sample added was shaken in an air bath at 37°C for 2 h, then centrifuge at 3525 × g for 20 min. Aspirate 1 ml of supernatant and added 6 ml of H_2_SO_4_ (45%) and 1 ml of furfural (0.3%) and left in a water bath at 65°C for 30 min, and the unbinding cholate in the supernatant was determined at 620 nm with a spectrophotometer (UV-1500PC, Shanghai Meisei Instruments Co, Shanghai City, China.).

#### 2.8.3. Nitrite adsorption capacity

The nitrite adsorption capacity of mung bean flour was determined according to the method of Li et al. (29) with some modifications. 1 g sample and 30 ml 50 μg/ml NaNO_2_ was taken, adjusted pH = 2, shaken for 2 h at 37°C in an air bath, and then centrifuged at 4000 rpm for 20 min. Aspirate 1 ml of supernatant, add 2 ml of p-aminobenzenesulfonic acid (4 mg/ml) and let stand for 5 min. Continue adding 1 ml of N-Ethylenediamine dihydrochloride (2 mg/ml) and let stand for 15 min. The unbinding nitrite in the supernatant was determined at 538 nm with a spectrophotometer.

### 2.9. Statistical analysis

All measurements were performed in triplicate. Data analysis was performed using SPSS (version 22, IBM, Armonk City, New York, NY, United States) and Origin software (version 9.1, OriginLab, Northampton, MA, United States). The data were expressed as mean ± standard deviation and the difference was at the 95% level of significance (*p* < 0.05) using one-way ANOVA.

## 3. Results and discussion

### 3.1. Morphological properties

**Figure 1:**
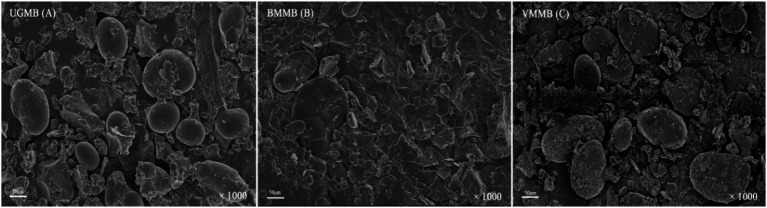
Scanning electron micrographs of different mung bean flour UGMB **(A)**; BMMB **(B)**; VMMB **(C)**.

The scanning electron micrographs of mung bean flour produced by three kinds of milling methods are shown in Figure 1. The UGMB exhibited an ovoid or irregular shape, and some of the particles were damaged. However, the overall particle structure was relatively intact (Figure 1A). This result was in line with the findings of Yu et al. (19), who observed the morphology of buckwheat flour produced by three different grinding methods. Compared to UGMB, the morphology of BMMB and VMMB (Figures 1B,C) was significantly changed. BMMB and VMMB particles were broken to a large extent, while some particles were deformed. However, VMMB changed the particle shape from ovoid to flattened due to the strong impact. Moreover, UGMB particles were in a loose state, whereas BMMB and VMMB showed particle aggregation, which might be due to the electrostatic force generated during the high-speed grinding process, making small particles adhere to the large particle. Zhang et al. (36) found that ultrafine grinding could lead to a trend of first decreasing and then increasing the average height of the spherical structure of LBP, which they suggested might be related to the van der Waals and electrostatic forces generated by the high-speed shearing process during comminution, and the phenomenon of inter/intra-molecular aggregation. In addition, results consistent with this paper were obtained by Yang (37) and Chen et al. (38). These results indicated that ball and vibratory mills could significantly increase the breakage capacity of particles compared to universal mills.

### 3.2. Particle size distribution analysis

**Figure 2:**
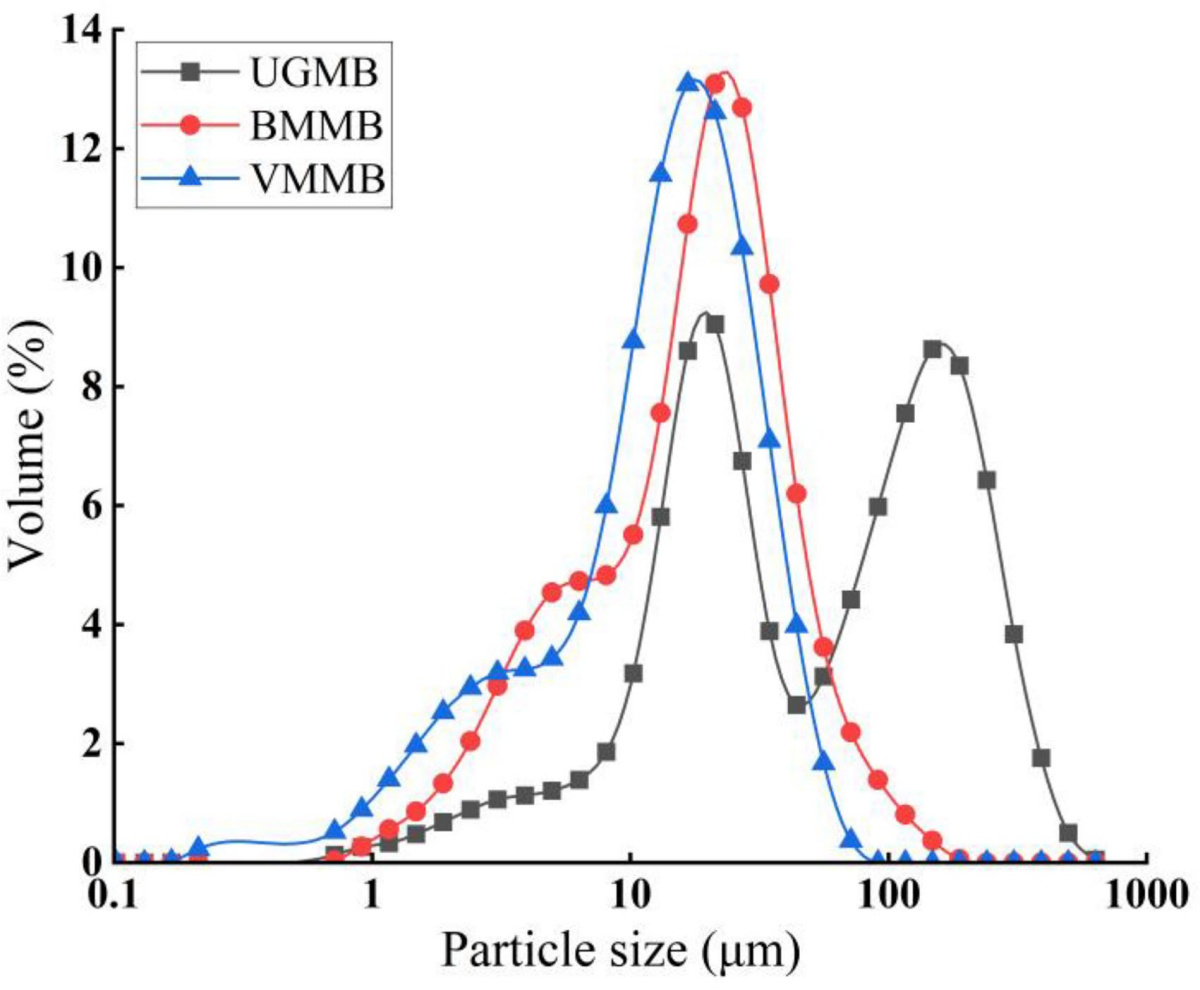
Particle size distribution by volume of mung bean flour.

The particle size distributions of different mung bean flour samples are displayed in Figure 2. The particle distribution is expressed as the percentage of particles in the total dust. BMMB and VMMB showed the same trend, whereas UGMB samples displayed a distinct bimodal distribution. For different milling methods, the D_50_ values of the UGMB, BMMB, and VMMB samples were 59.34, 21.34, and 26.55 μm, respectively, with specific surface areas of 0.097, 0.180, and 0.158 m^2^/g (Table 1). Due to the difference in mechanical force of grinding process, the BMMB showed the smallest D_50_ value, while the largest D_50_ value was obtained for UGMB. Moreover, the specific surface area tended to increase as the particle diameter decreased, indicating that ball milling and vibratory milling can reduce the particle size of mung bean flour to a greater degree and produce more uniformly distributed particles, which was further confirmed by the particle size distribution curves. This phenomenon also appears in the results of Yu et al. (19), which investigated the effect of three different grinding methods on the particle size distribution of buckwheat flour, and obtained the conclusion that the buckwheat flour treated with wet grinding and graphite has a smaller particle diameter and more uniform distribution than the normal crushing treatment, which is consistent with the results of this study.

**Table 1:** Particle size distribution, specific surface area, particle density and colour.

Samples	D_50_ (μm)	S_w_ (m^2^/g)	Td (kg/cm^3^)	Pd (kg/cm^3^)	Colour parameter		
					L*	a*	b*
UGMB	59.34 ± 1.75^a^	0.097 ± 0.02^c^	0.93 ± 0.01^a^	0.69 ± 0.01^a^	82.96 ± 0.57^c^	−3.41 ± 0.05^c^	18.51 ± 0.18^a^
BMMB	21.34 ± 0.29^c^	0.180 ± 0.03^a^	0.72 ± 0.01^b^	0.42 ± 0.01^b^	90.80 ± 0.10^a^	−1.64 ± 0.02^a^	9.84 ± 0.11^c^
VMMB	26.55 ± 0.25^b^	0.158 ± 0.01^b^	0.69 ± 0.01^c^	0.41 ± 0.01^b^	87.85 ± 0.14^b^	−2.76 ± 0.11^b^	12.86 ± 0.25^b^

D_50_ stands for the median particle size; S_W_ stands for specific surface area; Td stands for Tap density; Pd stands for packing density; L^*^, a^*^, b^*^ represent L^*^ + means whiter and L^*^-means darker; a^*^ + means red a^*^-means green; b^*^ + means yellowish, b^*^-means bluish. Data are expressed as the mean ± standard deviation. Values in the same column with different letters are significantly different at *p* < 0.05.

### 3.3. Particle density (td and Pd)

The effects of different grinding methods on the particle density of mung bean powder are shown in Table 1. There is a significant difference in particle density between UGMB, BMMB, and VMMB. The tap densities of BMMB and VMMB were 0.72 and 0.69 kg/cm^3^, respectively, which decreased by 32.6 and 25.9% compared to UGMB. The packing density of BMMB and VMMB were 0.41 and 0.42 kg/cm^3^, respectively, which decreased by 39.2 and 40.6% compared to UGBM. These results showed that particle density decreased with decreasing particle size. In the high-speed shear process, the particles will be with the crushing cavity and particles between each other friction, the electrostatic force and van der Waals force resulting in smaller particles attached to the surface of larger particles (Figure 1), the emergence of this condition to increase the gap between the powder. Simply put, the particles are loosely packed with each other, and the density of particles becomes larger. This phenomenon is more obvious after ultrafine grinding. This trend is consistent with the results of Wang et al. (39) and Syahrizal et al. (40).

### 3.4. Measurement of color

**Figure 3:**
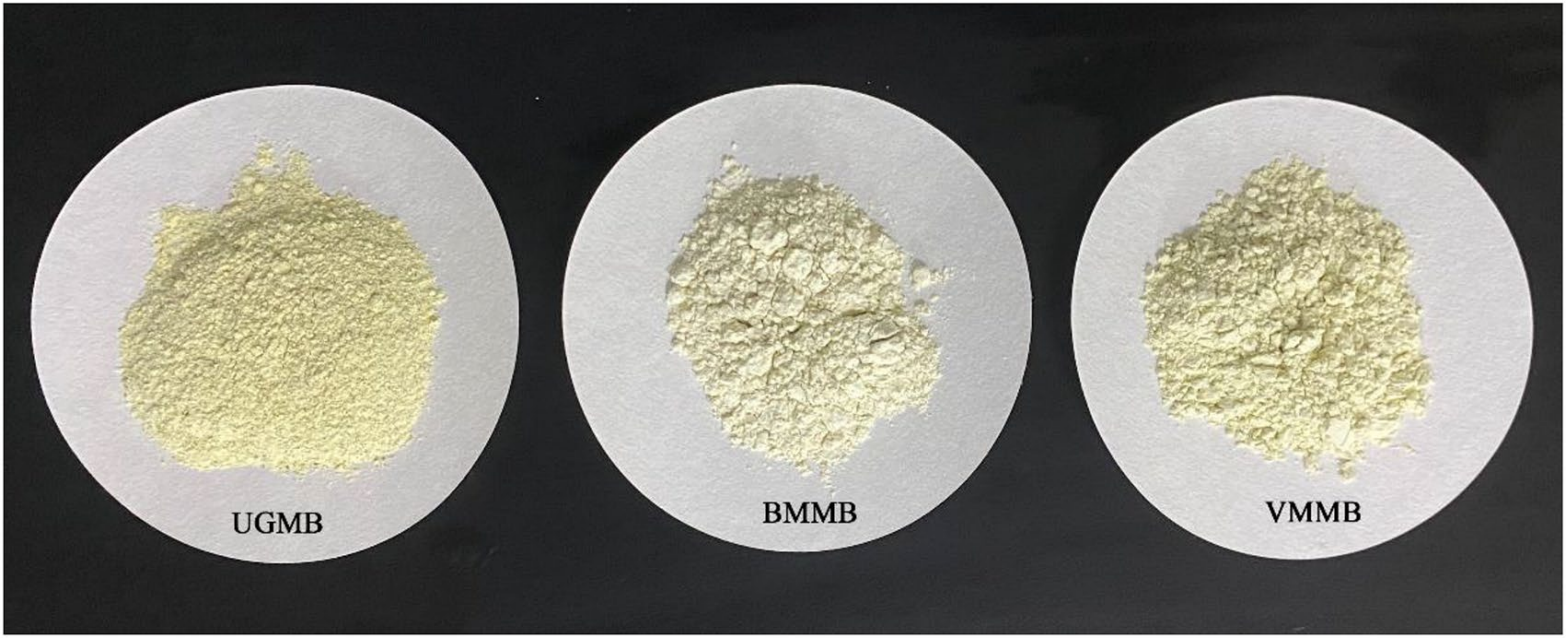
Morphology of mung bean powder under different grinding methods.

The different L*, a*, and b* values for mung bean flour are shown in Table 1 and Figure 3. Compared to UGMB, L* and a* values increased and b* values decreased in BMMB and VMMB; the difference between the values was significant (*p* < 0.05). L*, a*, and b* values of 82.96, −3.41, and 18.51 for UGMB and increased L* values of 90.80 and 87.85 for BMMB and VMMB, respectively, indicated an increase in the color brightness of the samples. The a* values increased to –1.64 and –2.76, indicating a decrease in greenness while the b* values decreased to 9.84 and 12.86, indicating a decrease in yellowness. The increase of L* and a* proved that the unbreakable mung bean seed coat of the sample was damaged to a greater extent after the ultra-fine grinding treatment. The seed coat particle size decreased, resulting more uniform distribution in the mung bean powder. The overall color of mung bean powder was more uniform, the brightness was improved, and the green color of mung bean powder was reduced due to the uniform distribution of the seed coat. Compared with VMMB, the L* and a* values of BMMB appeared to be greater, proving that ball mill comminution could result in a more uniform powder distribution.

### 3.5. Hydration properties (WHC and OHC, S and SP)

The WHC, OHC, S, and SP of mung bean flour are shown in Table 2. The WHC of UGMB, BMMB, and VMMB was 3.12, 3.33, and 3.62 g/g, respectively. The WHC of the samples subjected to ultrafine grinding showed an increasing trend, with WHC increasing 6.7–16.1%. OHC showed the same tendency as WHC. Compared with UGMB, the OHC of BMMB and VMMB was also improved by 5.1–11.3%. The results of this study contradicted the findings of Gong et al. (41) which reported that WHC and OHC decreased with decreasing particle size by ultrafine grinding of processed mushroom powder. The reason might be that the high starch and protein content in mung beans increases the degree of particle breakage under the action of powerful mechanical force of ultrafine grinding crushing, and the large particles are broken down into small particles (Figure 1), which increases the area of the internal structure of mung beans with the release of water and oil. As the particle size decreases, the WHC and OHC capacity increases. Compared with VMMB, the WHC and OHC values of BMMB were slightly lower, indicating that the mechanical forces generated by the vibratory mill treatment were stronger than those of the ball mill treatment.

**Table 2:** Water holding capacity, oil holding capacity, solubility and swelling power.

Samples	WHC (g/g)	OHC (g/g)	S (%)	SP (%)
UGMB	3.12 ± 0.11^c^	1.95 ± 0.01^c^	0.39 ± 0.14^a^	0.83 ± 0.23^b^
BMMB	3.33 ± 0.03^b^	2.05 ± 0.02^b^	0.35 ± 0.02^b^	0.86 ± 0.12^a^
VMMB	3.62 ± 0.14^a^	2.17 ± 0.02^a^	0.36 ± 0.19^b^	0.87 ± 0.16^a^

WHC stands for water holding capacity; OHC stands for oil holding capacity; S stands for solubility; SP stands for swelling power. Data are expressed as the mean ± standard deviation. Values in the same column with different letters are significantly different at *p* < 0.05.

The S of BMMB and VMMB was reduced compared to UGMB. The S of BMMB and VMMB decreased by 10.3 and 7.7%, respectively. After the superfine grinding of mung bean flour, the branch chain of branched starch was broken, and the straight chain starch content increased due to the strong mechanical force (42). Branched starch molecules are more likely to form hydrogen bonds with water molecules, whereas straight-chain starches are generally closely packed and curled, easily forming intramolecular hydrogen bonds, and preventing water molecules from entering. The SP exhibited an opposite to S. The SP of BMMB and VMMB increased by 3.5–4.6%. The increase in straight-chain starch content resulted in an increase in the internal spatial dislocation effect of starch molecules, which disrupted the ordered structure and loosened the structure, hence contributing to its water absorption and swelling. Sharma et al. (43) were consistent with the results of this study.

### 3.6. Pasting properties

**Figure 4:**
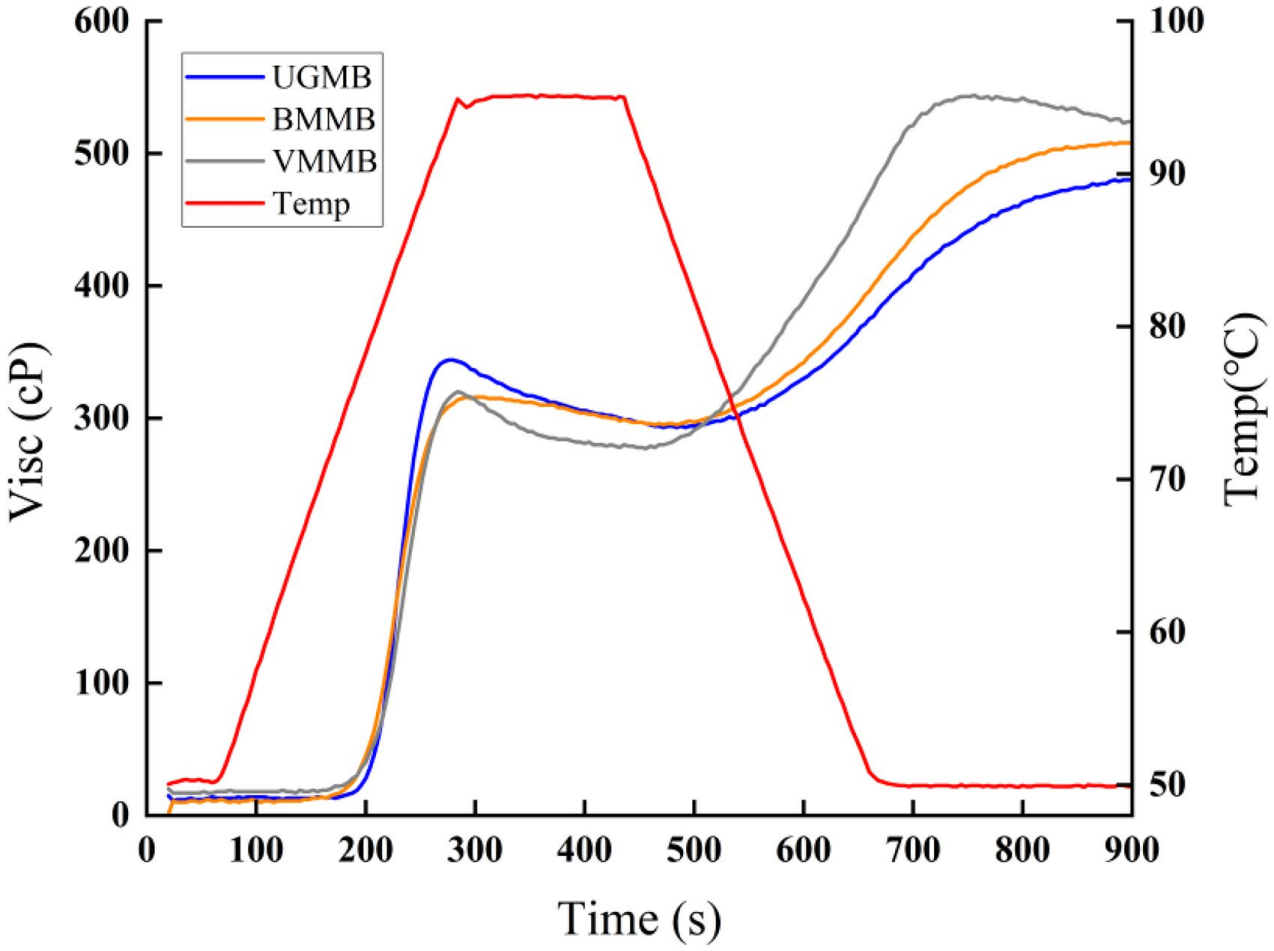
Viscosity curve of mung bean flour.

The RVA curves of UGMB, BMMB, and VMMB are shown in Figure 4 and Table 3. As shown in the figure, the shapes of pasting curves were similar for all mung bean flour samples, but the pasting parameters differed significantly. From the table, it can be seen that UGMB has the highest peak viscosity and pasting temperature. Highest BMMB valley viscosity, VMMB has the highest final viscosity. Peak viscosity is defined as the maximum viscosity reached when heating the powder slurry in the RVA curve, where the starch granules swell to their maximum extent. The higher peak viscosity in UGMB is consistent with the study of Wang and Xiao (44) who reported higher peak viscosity in the paste profile of air-pulverized corn starch. The results may be attributed to the high swelling capacity (Figure 2). The differences in viscosity of mung bean flour may be attributed to differences in straight-chain starch leaching and granule swelling (45). The final viscosity indicates the ability of the material to form a sticky paste and is mainly determined by the regeneration of soluble straight-chain starch during cooling (46). The lowest attenuation of UGMB indicates that UGMB has good anti-aging properties and can form stable pastes. The results of this study are in agreement with Yu et al. (19).

**Table 3:** Pasting characteristics parameters of mung bean flour.

Sample	Peak viscosity (cP)	Valley viscosity (cP)	Final viscosity (cP)	Pasting Temp (°C)
UGMB	344 ± 3^a^	293 ± 3^b^	480 ± 2^a^	84.32 ± 0.3^a^
BMMB	316 ± 4^b^	295 ± 6^a^	508 ± 2^c^	80.70 ± 0.4^c^
VMMB	320 ± 3^c^	279 ± 4^b^	524 ± 4^b^	82.25 ± 0.3^b^

Data are expressed as the mean ± standard deviation. Values in the same column with different letters are significantly different at *p* < 0.05

### 3.7. Thermal properties

**Figure 5:**
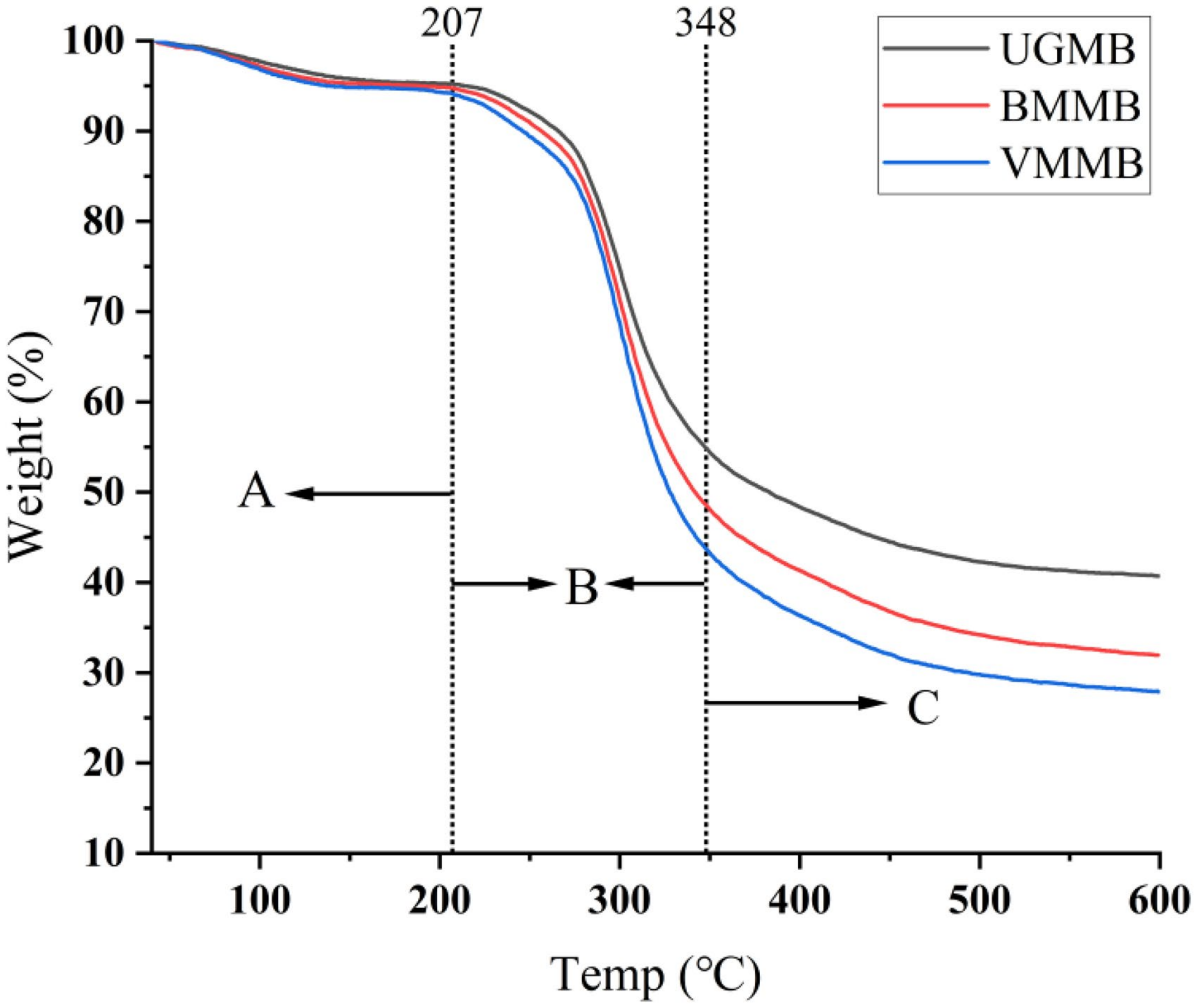
Thermal curve of mung bean flour: **(A)** is the water evaporation stage, **(B)** is the rapid pyrolysis stage, **(C)** is the carbonization stage.

The TG curves of UGMB, BMMB, and VMMB are shown in Figure 5. In general, the moisture evaporation order, the rapid pyrolysis stage, and the carbonization stage together form the thermal decomposition process. Information from Figure 5 indicated that the three samples were subjected to a water evaporation phase from room temperature to 207°C, and the TG curves at this stage showed a slight weight loss of the system. The sample mass decreased rapidly in the range of 207–348°C. This was the main stage of combustion within the system, with a weight loss rate of about 50–60%, primarily due to the destruction of the starch chain structure within the system. The carbonization stage was in the range of 348–600°C, leading a reduction in sample mass due to the thermal decomposition and carbonization of starch, protein, and dietary fiber in the system. The TG intervals of UGBM, BMMB, and VMMB overlapped during the water evaporation phase. The weight loss of BMMB and VMMB decreased significantly during the evaporation phase. Therefore, we speculate that the possible reason is that the ultrafine grinding crushing destroys the sample particles and reduces the crystallinity, so that more bound water becomes free water and cannot effectively resist the thermal decomposition effect.

### 3.8. Rheological properties

**Figure 6:**
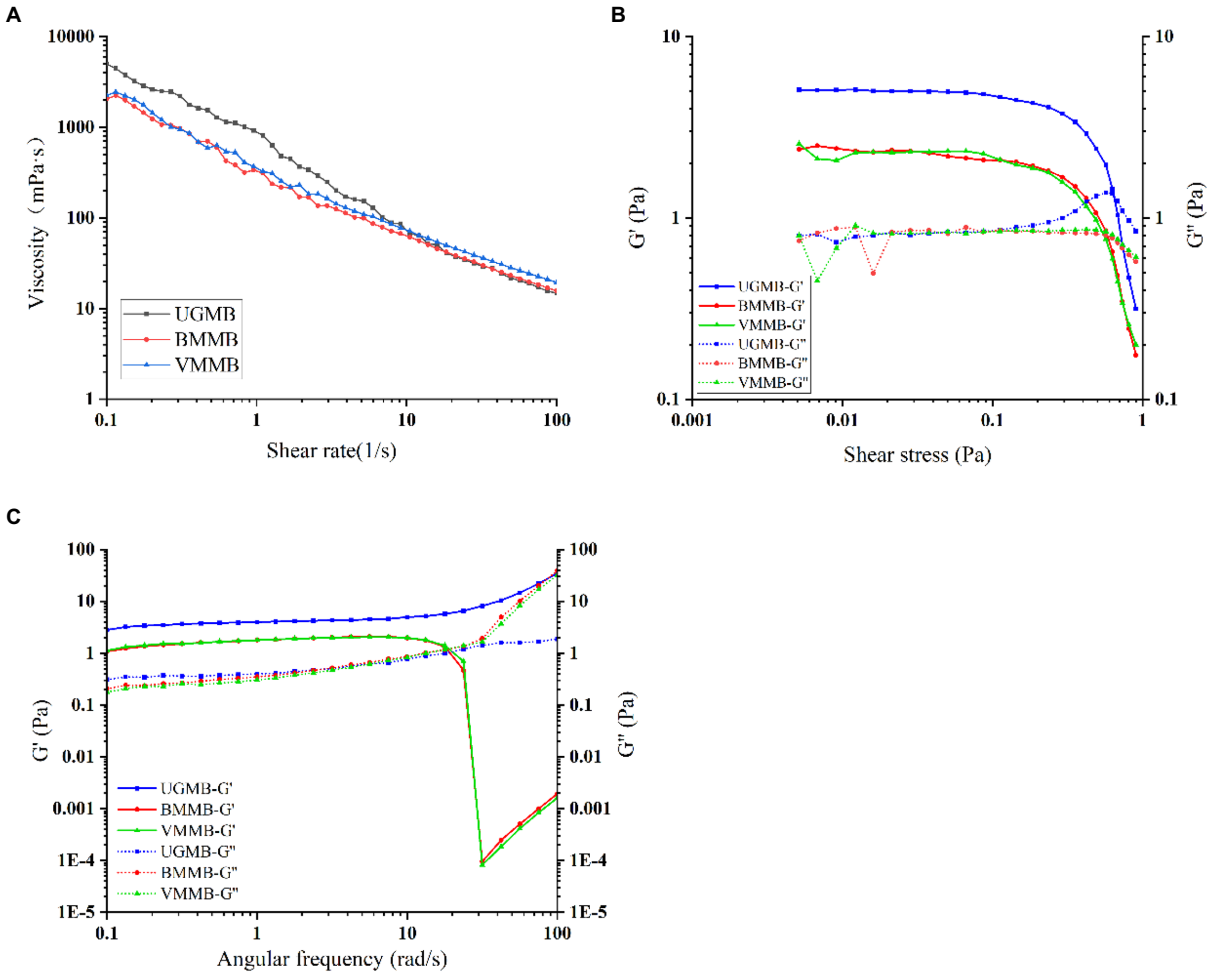
Rheological curve of mung bean flour.

The UGMB, BMMB, and VMMN rheological curves were fitted using the power law equation *τ* = *Kγn*. According to the Table 4, the flow coefficients of the model ranged from –0.7973 ~ −0.8324 (*n* < 1), reflecting the pseudoplastic characteristics of the sample, and demonstrating that the sample conforms to the non-Newtonian flow law. As shown in Figure 6A, the viscosity of the treated samples (BMMB and VMMB) was lower than that of UGMB. Due to water absorption and expansion, starch molecules cross-linked to form a gel state. When the role of shear stress increased, the system of mutually cross-linked structure was straightened, and the physical cross-linking point destruction rate exceeded the reconstruction rate, leading to drop in the viscosity, resulting the phenomenon of shear thinning (47). Strong mechanical forces significantly damaged BMMB and VMMB particles, molecular chains were broken, loosened the structure, and the viscous resistance from convection was reduced, resulting in a viscosity lower than of UGMB.

**Table 4:** Mung bean powder rheological parameters.

Sample	Consistency factor (*K*)	Flow coefficient (*n*)	Decision factor (*R*^2^)
UGMB	773.8 ± 12.4^a^	−0.7973 ± 0.097^b^	0.9942 ± 0.002^a^
BMMB	340.5 ± 11.3^c^	−0.8324 ± 0.076^a^	0.9891 ± 0.003^b^
VMMB	389.0 ± 13.2^b^	−0.7992 ± 0.105^b^	0.9815 ± 0.002^b^

Data are expressed as the mean ± standard deviation. Values in the same column with different letters are significantly different at *p* < 0.05.

As it can be seen from Figure 6B, the yield stresses of BMMB and VMMB decreased as compared to UGMB. The treated samples (BMMB and VMMB) increased straight-chain starch content, weakened the binding capacity to water, disrupted the gel network structure, decreased the resistance to shear stress within the molecule, and reduced yield stress. The G″ values of the three samples largely overlapped within the linear viscoelastic region, while the G′ of the treated samples (BMMB and VMMB) was lower than that of UGMB, indicating a decrease in elasticity.

As shown in Figure 6C G′ was significantly higher than G″ between the systems in the frequency sweep range (0–17.8 rad/s). Except for UGMB, both G′ and G″ of BMMB and VMMB seemed to be crossed. When the frequency scan value was v 17.8 rad/s, both BMMB and VMMB showed the phenomenon that G’ was much smaller than G″ indicating viscous deformation within the system and the rapid transition of morphology from gel to liquid. In the entire frequency range, the gel strength of UGMB was stronger than that of BMMB and VMMB, indicating that it was more significant than BMMB and VMMB in terms of hardness.

### 3.9. Functional characteristics

**Figure 7:**
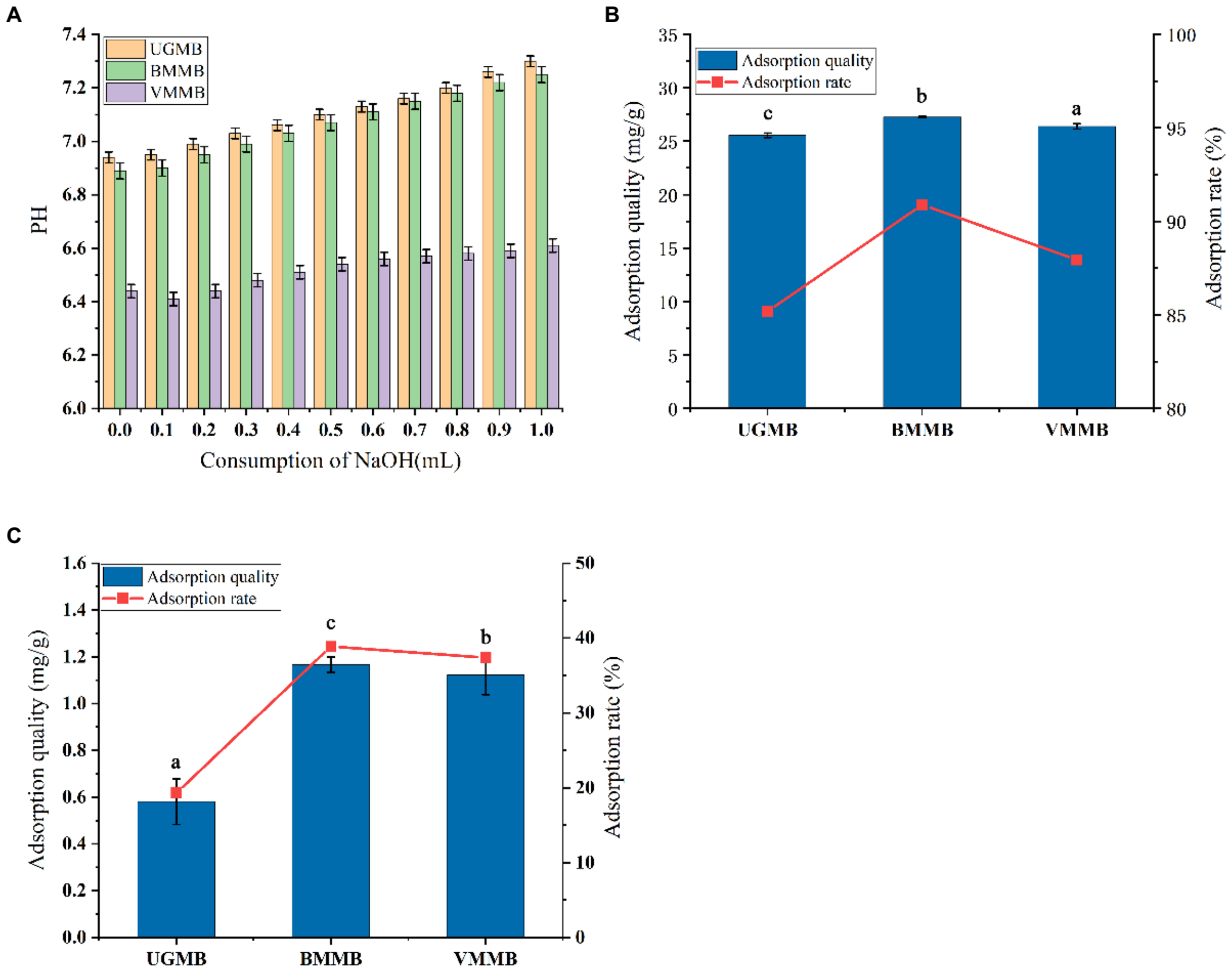
Functional characteristics of Mung bean powder: **(A)** is the cation exchange capacity, **(B)** is the adsorption capacity of cholate, and **(C)** is the adsorption capacity of tobacco nitrate.

The cation exchange capacity of mung bean powder is shown in Figure 7A. The cation exchange capacity relates to the breakage of fiber chains and the degree of exposure of groups; the more exposed groups, the more favorable the adsorption of harmful substances is. With the continuous addition of NaOH to the solution, the pH of the sample solutions treated by the three crushing methods showed the same increase, but the rate of increase varied significantly. When the total amount of NaOH in the solution was increased to 1 ml, the exchange capacity of UGMB (pH = 7.30) cations was significantly lower than that of BMMB (pH = 7.25) and VMMB (pH = 6.58) This was primarily caused by the increase in side chain groups required for the exchange reaction to occur, the disruption of the fiber mesh structure by ultra-micro-grinding, and the stronger the degree of comminution and the mechanical force of comminution, exposing more groups in the molecule. VMMB and BMMB exposed more groups and bound the OH-in solution more effectively, therefore the solution pH appeared to rise slowly. The study of Huang et al. (48) described the effect of ultra-fine grinding on the cation exchange capacity of wheat bran diet, and the results were similar to those discussed in this paper.

The cholate adsorption capacity of mung bean powder is shown in Figure 7B. The cholate adsorption capacity is influenced by the degree of effective bile acid salt binding in the dietary fiber body, inhibiting the absorption of fat from dietary intake. The adsorption amounts of UGMB, BMMB, and VMMB were 25.56 mg/g, 27.27 mg/g, and 26.38 mg/g. When compared to UGMB, the samples obtained by ultra-fine grinding treatment (BMMB and VMMB) showed different degrees of enhancement in their binding capacity to bile acid salts, with BMMB showing the best binding capacity and VMMB slightly lower than BMMB. Ball milling and vibratory crushing processes have the potential to affect the particle integrity to a greater extent, and the formation of pores between the broken particles can effectively bind and hold the bile acid molecules. In summary, the binding ability of mung bean flour to bile acid salts was mainly due to the adsorption and wrapping ability of dietary fiber. Ultrafine grinding grinds the dietary fiber in whole seed mung bean flour into finer and more uniform particles, allowing it to bind bile acid salts more efficiently. Zhu (49) results are similar with this paper.

The nitrite adsorption capacity of mung bean powder is shown in Figure 7C. NO2-in the body can react with secondary and tertiary amines to produce carcinogenic substances, nitrosamines, which have various toxic effects on the human body, but the intake of a certain amount of dietary fiber can usually inhibit the toxicity of nitrite after excessive intake Zhu (49). Comparing the binding capacity of UGMB, BMMB, and VMMB to NO^2−^ in solution, BMMB had the best capacity with 38.87% adsorption while VMMB (37.39%) was slightly lower than BMMB. Both significantly improved as compared to UGMB (19.34%). This phenomenon may be due to the fact that ultrafine pulverization destroys the original structure of the particles and converts the insoluble dietary fiber in the system into soluble dietary fiber containing glyoxalate. Under acidic conditions NO^2−^ can combine with H+ to form HNO_2_, which further accumulates in solution to form the electrophile NaNO_3_, which can effectively bind to the negatively charged oxygen atoms on the phenolic acid groups in dietary fibers, thus acting as an adsorbent (50, 51).

## 4. Conclusion

The effects of different drying and crushing methods on the physicochemical properties of whole grain mung bean flour were investigated. Due to the different grinding methods, the physicochemical properties of the samples were significantly different. Superfine crushing resulted in a significant reduction in the particle size of mung bean flour, reflecting changes in the structural and functional properties of microfined mung bean flour. After the ultrafine grinding crushing treatment, the surface of mung bean flour particles become rough and the average particle size is reduced. In addition, the WHC, OHC and final viscosity were significantly higher than those of coarse mung bean flour after ultrafine grinding. The rheological and functional property tests showed that superfine crushing significantly reduced the yield stress of mung bean flour and significantly increased the adsorption capacity of bile acid salt and nitrite, and enhanced the cation exchange capacity. The structural and functional properties of micronized mung bean powder are closely related to the crushing method. The method and choice of comminution is a key factor affecting the structural, physicochemical and functional properties of the powder. The potential applications and uses of ultrafine grinding comminution modification in the food industry, such as as a textural improver in mixed grain bread mixes, need further investigation.

## Data availability statement

The original contributions presented in the study are publicly available. This data can be found here: https://figshare.com/s/a499fd6b6672ae178047.

## Author contributions

SY carried out the experimental work and data analysis. SY and YW drafted the manuscript. ZL and CW conceptualized the study. DZ and LW supervised the study and critically revised the draft. All authors contributed to the article and approved the submitted version.

## Funding

The work was supported by the National Key Research and Development Program of China (2021YFD2100902); the authors gratefully acknowledge the financial support provided by the Natural Science Foundation of Heilongjiang Province (LH2020C087); the Major science and technology projects of Heilongjiang Province (2021ZX12B0203); the Major science and technology projects of Heilongjiang Province (2021ZX12B06); the Special projects for the central government to guide local scientific and technological development (DQKJJYD0001).

## Conflict of interest

The authors declare that the research was conducted in the absence of any commercial or financial relationships that could be construed as a potential conflict of interest.

## Publisher’s note

All claims expressed in this article are solely those of the authors and do not necessarily represent those of their affiliated organizations, or those of the publisher, the editors and the reviewers. Any product that may be evaluated in this article, or claim that may be made by its manufacturer, is not guaranteed or endorsed by the publisher.
